# Supplementary material for: Learning curve and surgical outcome of robotic assisted colorectal surgery with ERAS program
Source: Sci Rep. 2022 Nov 29;12:20566. doi: 10.1038/s41598-022-24665-w (PMC9709162; doi:10.1038/s41598-022-24665-w)
Supplement: Supplementary file 2 — Supplementary Information 2. [file 41598_2022_24665_MOESM2_ESM.zip › RAL Raw data-Ñ[▒K/096/OP_ñw╝╨░O▒Kñσ.pdf]

正式摘要(電子簽章：000000220497134)

請選擇...reporter

臺 中 榮 民 總 醫 院

Operation Note

[redacted]

- 手術日期：2021/07/08
- 手術醫師：VS 蔣鋒帆，CR 楊曙榮，CR 陳映菁，R 郭連雄，R 陳映晴
- Anesthesia：GE
- Position：Lithotomy
- Aseptic agents：Soap，Betadine Alcohol，Betadine Aqua

Diagnosis

- Pre-operative diagnosis : Malignant tumor of rectum + T COLON CA
- Post-operative diagnosis : Ditto.

Surgical Indication

- Tumor resection

Operation

- Robot Assisted Low Anterior Resection

Finding

- 1. One ulcerative tumor about 6 cm in size at rectum 5 cm FAV, and one polypoid tumor about 8cm in size at distal T-colon.
  2. Total meso excision was complet
  3. IMA high ligation+
  4. distal margin 2 cm, Surefrom 45 x2 for rectum
  5. air tie test neg. donut intact. penrose in pelvis.
  6. No ileostomy due to patient insist
  7. Severe adhision around RLQ with mesocolon, T colon, A colon, small intestin and mesentery

Procedure

- 1. under GE, the pt was in lithotomy position
  2. the trocar hold was made in periumbilicus open method. the other 5 hole made by punctiure
  3. identify holly plain and dissct. isolated IMA root. ligated with clips
  4. dissect between Gerota fascia and meso rectum. lysis Lt white line
  5. dissect into pelvis area. dissect posterior side of rectum down to pelvis follor levetar ani. anterior side of Denovillor fascia. Total meso excision was complet
  6. resected rectum stump with Sure Form 45 2 fires.
  7. We change to laparoscopy and one 5mm trochar made at LLQ
  8. Lysis was done and the splenic flexure and hepatic flexute was taken down
  9. pull the specimen outside abdomen from midline wd
  10. resect laparoscopy
  11. embeded envril 33 mm
  12. complete anastomsis. air tie test neg. donut intact. penrose in pelvis.
  13. No ileostomy due to patient insist
  14. She tolerate whole procedure and extubated.
- Estimated blood loss: minimum
- 器械數量: 正確
- 紗布數量: 正確

Image

- PAINT
    - abdomen

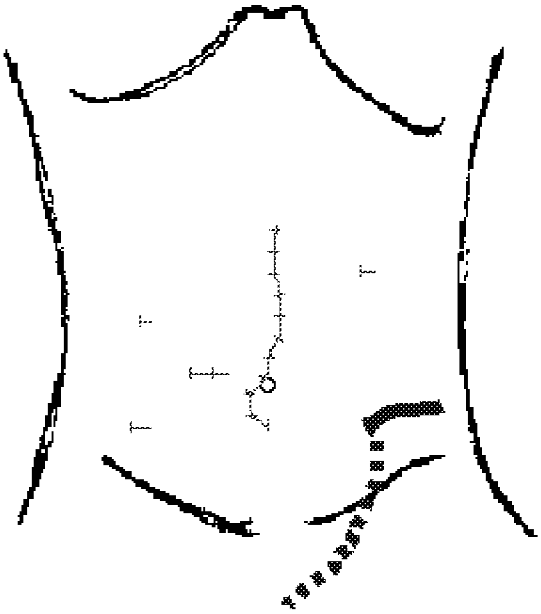

    - colon & rectum

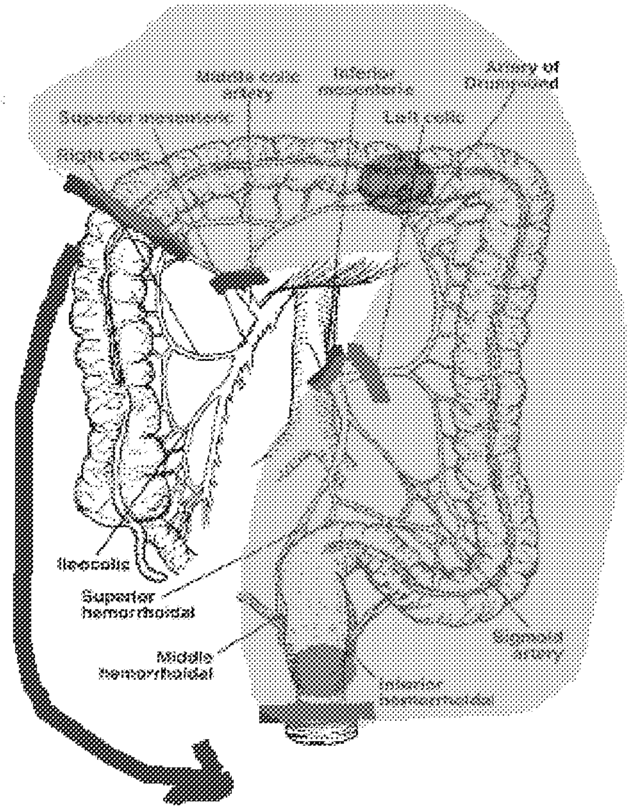

主任 2062F蔣鋒帆 / 醫師 2292G楊曙榮

正式摘要(**電子簽章：000000220497134)**

請選擇...reporter

doctorRank:B
